# The Effectiveness of the Kangaroo Care Method in Neonates and Its Relationship With Parental Stress and Satisfaction in the Neonatal Intensive Care Unit

**DOI:** 10.7759/cureus.106863

**Published:** 2026-04-11

**Authors:** Ilias Kapsiotis, Varvara Boutopoulou, Anastasia Kapetanaki, Iraklis Salvanos, Vasiliki Matziou

**Affiliations:** 1 Obstetrics and Gynecology, "Elena Venizelou" General Hospital - Maternity Hospital, Athens, GRC; 2 Nursing, National and Kapodistrian University of Athens, Athens, GRC; 3 Pediatrics, "Elena Venizelou" General Hospital - Maternity Hospital, Athens, GRC

**Keywords:** kangaroo care, nicu, parental satisfaction, parental stress, skin-to-skin contact

## Abstract

Introduction

Kangaroo care (KC), defined as the first, sustained skin-to-skin contact between parents and their neonates, is widely recognized as a key family-centered intervention in the Neonatal Intensive Care Unit (NICU). Beyond its physiological characteristics, including improved thermoregulation, cardiorespiratory stability, and breastfeeding, KC has demonstrated significant psychosocial benefits, particularly in reducing parental stress and enhancing parent-neonate bonding. Despite strong international evidence, there is limited data from the Greek NICU setting on the association of KC with parental stress and satisfaction. This study aimed to evaluate the effect of KC in reducing parental stress and increasing parental satisfaction with neonatal care.

Methods

A quasi-experimental two-group design (intervention versus control) was implemented in a Greek NICU. A total of 80 parents of preterm or full-term neonates (gestational age 24-40 weeks) participated. Parents in the intervention group (n=40) applied KC for 1 hour daily throughout the hospitalization, while the control group (n=40) received regular care with limited physical contact. Parental stress was assessed using the Parental Stress Scale: NICU (PSS: NICU) at baseline and at the end of hospitalization, and parental satisfaction using the NICU Parent Satisfaction Form (NICU-PSF). The clinical status of the neonate was assessed using the ALPS-Neo scale. Independent t-tests and Chi-square tests were used for comparisons between groups, and Spearman’s rho correlations examined associations between demographic/clinical variables and parental outcomes.

Results

Parents in the KC group showed significantly lower total scores compared to controls (107.69 ± 9.45 vs. 121.40 ± 14.68, p = 0.015). Stress related to the change in parental role was also significantly reduced (32.92 ± 2.89 vs. 38.60 ± 6.08, p = 0.002). No significant differences were observed in stress related to NICU sounds (p = 0.46) or the appearance of the newborn (p = 0.11). Overall parenting satisfaction was high in both groups (KC group: 4.14 ± 0.44 vs control group: 4.18 ± 0.02, p=0.572). Maternal age was negatively associated with anxiety across all domains (rho = -0.337 to -0.389, p < 0.01). Perceived family support showed a strong positive association with anxiety related to the NICU environment and neonate appearance (rho = 0.61-0.73, p < 0.001). Neonatal clinical status (ALPS-Neo) was positively associated with both parenting satisfaction (rho = 0.473, p < 0.001) and stress related to neonate appearance (rho = 0.383, p = 0.001).

Conclusion

KC was associated with significantly lower parental stress, particularly regarding parental role disruption, reinforcing its value in enhancing parental involvement and emotional well-being in the NICU. Parental satisfaction remained high in all groups, further influenced by the clinical status of the neonates. The findings support the extensive, systematic integration of KC in Greek NICUs, aligned with international recommendations, to improve both parental psychological outcomes and overall quality of neonatal care.

## Introduction

The care of the neonates hospitalized in neonatal intensive care units (NICUs) represents one of the most demanding, multifaceted, and ethically sensitive areas of healthcare, as it integrates advanced medical technology, highly specialized nursing expertise, and the imperative for a family-centered and developmental care approach [1]. The hospitalization of a neonate in the NICU is a deeply distressing and emotionally taxing experience for parents, who often report overwhelming feelings of fear, helplessness and detachment from their parent role, largely due to the technologically intensive environment and the limited opportunities for physical contact with their neonate [2,3]. Within this context, the kangaroo care (KC) method has emerged internationally as a simple, evidenced-based and cost-effective intervention that significantly promotes the physiological stability and psycho-emotional well-being of both neonates and the parents [4].

The KC approach is based on direct skin-to-skin contact between the neonate and the parent - most commonly the mother, though increasingly also the father - with the infant positioned against the caregiver’s bare chest. This practice is applicable to both preterm and full-term neonates, who are in stable or semi-stable clinical condition and is frequently combined with exclusive breastfeeding and earlier discharge from the NICU [5]. A meta-analysis conducted by Boundy et al., encompassing 124 studies, demonstrated that KMC significantly reduces neonatal mortality, sepsis, and hypothermia, while simultaneously enhancing weight gain, thermoregulation, and breastfeeding rates [6]. More recent evidence from the WHO Immediate KMC Study Group (2021) emphasized that initiating KC immediately after birth - even before fully stabilization of the neonate - can reduce mortality by up to 25%, underscoring its life-saving potential in both high-and low-resource settings [7].

Beyond its well-established physiological benefits, KC holds profound psychological, social, and developmental significance, as it fosters secure parent-neonate bonding, alleviates parental stress and anxiety, and strengthens parents’ sense of competence, empowerment, and caregiving identity [8,9]. The prolonged physical proximity achieved through KC is believed to activate neuroendocrine mechanisms that decrease cortisol levels, increase oxytocin release, and induce a state of psychophysiological calm in both the neonate and the caregiver [10]. For parents who often perceive the NICU as a technologically alienating environment, active participation through KC serves as a bridge between biomedical interventions and natural human caregiving, promoting emotional connection and family integration within intensive care [11].

At the same time, parental satisfaction has been recognized as a key indicator of quality, safety, and organizational effectiveness in the management and evaluation of healthcare services. Within the framework of family-centered care, parental satisfaction extends beyond the technical adequacy of medical treatment to encompass respectful communication, empathy, partnership, and emotional support [12,13]. Evidence indicates that higher levels of parental satisfaction are associated with improved neonatal health outcomes, greater adherence to post-discharge instructions, stronger trust in healthcare professionals, and fewer conflicts between families and staff [14]. In this regard, interventions such as KC not only enhance clinical outcomes but also function as catalysts for organizational excellence and compassionate, family-integrated neonatal care.

Despite its well-documented benefits, the systematic evaluation of the KC in relation to parental stress and satisfaction with neonatal care remains notably limited, particularly within the Greek healthcare context. Addressing this gap is essential, as understanding the psychosocial dimensions of KC can inform both clinical practice and organizational strategies aimed at enhancing family-centered care in the NICU setting.

The present study, therefore, aims to evaluate primarily the effect of the KC among neonates hospitalized in the NICU and, secondly, to explore its association with parental stress and satisfaction with the care provided. It is hypothesized that implementation of KC will be significantly associated with lower levels of parental stress and higher levels of satisfaction, thereby contributing to the improvement of both the parental experience and the overall quality of neonatal healthcare services.

## Materials and methods

Study design

A quasi-experimental two-group design (interventions vs control) was implemented in a Greek NICU. Participants were allocated to the intervention and control groups using a non-random, convenience-based approach, based on clinical and organizational feasibility. The primary objective was to evaluate the effectiveness of the KC in reducing parental stress, and the secondary outcome was parental satisfaction. Data collection was conducted in the NICU of a Greek General and Maternity Hospital, following formal approval by the Scientific Council and the Hospital Ethics Committee. Data collection was conducted over a 12-month period, from November 2023 to November 2024.

Sample

The study population comprised parents of preterm and full-term neonates with a gestational age ranging from 24 to 40 weeks, all of whom provided written informed consent prior to participation.

The inclusion criteria for participation required that parents had sufficient proficiency in the Greek language and that their neonates had been hospitalized in the NICU for more than five days. Following eligibility confirmation, participants were randomly assigned to two groups. The intervention group (Group A) received KC, while the control group (Group B) received routine care, which consisted of a scheduled 30-minute visitation period with limited physical contact.

Intervention

Parents in the intervention group practiced KC for one hour daily throughout the neonate’s hospitalization, within the operational framework of the NICU. Although current WHO recommendations (2022) advocate early initiation and prolonged or continuous skin-to-skin contact (8-24 h/day) [15], the duration in this study was adapted to local clinical feasibility and parental availability.

All parents received standardized training by the principal investigator to ensure the proper and safe implementation of the method.

In the control group, the standard NICU routine was maintained, allowing brief scheduled visits and minimal physical interaction. 

During each session (either intervention or control), the neonates’ pain level was assessed using the Astrid Lindgren Children’s Hospital Pain Scale (ALPS-Neo), and their sleep-wake state was evaluated with the Neonatal Behavioural Assessment Scale (NBAS). Both measures were recorded at 10-minute intervals, and mean values per neonate were calculated for analysis.

Parents in both groups initially completed the Parental Stressor Scale: NICU (PSS: NICU) at two time points: a) baseline, within the first three days of NICU admission prior to the initiation of the intervention, and b) post-intervention, near the end of hospitalization. 

The NICU Parent Satisfaction Form (NICU-PSF) was completed on the day of the neonate’s discharge from the NICU to assess the parents’ overall satisfaction with the care provided during hospitalization.

Measurement tools

Parental Stressor Scale: NICU (PSS: NICU) [16]: This 46-item instrument evaluates four domains of parental stress: environmental stimuli (sounds and sights), neonatal appearance, alterations in parental role, and relationships with staff. It has been adapted in Greek by Malliarou et al. [17].

NICU Parent Satisfaction Form (NICU-PSF) [18]: A 44-item questionnaire comprising 40 closed and 4 open-ended questions assessing overall satisfaction, communication, parental participation and education. It has been adopted in Greek by Tsironi et al. [19].

Neonatal Behavioural Assessment Scale (NBAS) [20]: Assesses six neonatal sleep-wake states (deep sleep to crying) based on motor activity, respiration, eye movement, and responsiveness to stimuli.

Astrid Lindgren Children’s Hospital Pain Scale (ALPS-Neo) [21]: Evaluate neonatal pain through observation of facial expression, limb movement, respiratory pattern and muscle tone.

Statistical analysis

Data were analysed using IBM SPSS Statistics, Version 21 (IBM Corp., Armonk, NY, USA). Continuous variables were summarized as means ± standard deviation (SD), while categorical variables were expressed as frequencies and percentages.

Comparisons between the intervention and control groups were performed using the independent-samples Student’s t-test for continuous variables and the Chi-square (χ2) test, as appropriate for categorical variables. Associations between demographic/clinical variables and parental outcomes (stress and satisfaction) were examined using Spearman's rank correlation coefficient.

All statistical tests were two-tailed, and a p-value < 0.05 was considered statistically significant.

Ethical considerations

The study protocol was reviewed and approved by the Scientific Council and the Ethics and Deontology Committee of the hospital where the research was conducted, in accordance with institutional and national ethical standards. All participants received verbal and written information regarding the purpose, procedures, and voluntary nature of the study and subsequently provided written informed consent prior to participation.

Strict measures were taken to ensure anonymity and confidentiality of the collected data. Each participant was assigned a unique identification code, used consistently throughout all stages of data collection, entry, and analysis. All study documents and electronic data are securely stored in restricted-access, password-protected files and will be retained for a minimum of three years following study completion, in accordance with ethical research standards.

## Results

A total of 80 parents participated in the study, with 40 in the intervention group (KC) and 40 in the control group (routine care). Stress levels were assessed once, following the completion of the intervention.

The main maternal age in the total sample was 32.4 ± 5.8 years, with no statistically significant difference between groups. Most participants were mothers (87.5%), while the majority reported moderate to higher educational attainment. No significant differences were observed between groups with respect to natural characteristics, including gestational age, birth weight, and length of NICU stay (Table 1).

**Table 1:** Demographic and clinical characteristics of the sample. Independent-samples Student’s t-test was used for continuous variables and the Chi-square (χ²) test for categorical variables. All p-values refer to two-tailed tests, and statistical significance was defined as p < 0.05.

Variable	Intervention (n = 40)	Control (n = 40)	Test Statistic	p-value
Parent Demographics				
Parent gender (mother, %)	85	90	t=0.670	0.505
Maternal age (years), mean ± SD	32.1 ± 6.7	32.8 ± 5.6	t=-0.51	0.62
Paternal age (years), mean ± SD	32.7 ± 2.7	36.2 ± 7.6	t=-2.23	0.08
Previous NICU experience (%)	5.6	50	χ^2^=16.10	<0.001
Neonate Characteristics				
Sex (male, %)	52	48	χ^2^=0.00	0.79
Gestational age (weeks), mean ± SD	31.7 ± 0.97	29.6 ± 0.68	t=11.16	<0.001
Birth weight (g), mean ± SD	1581 ± 122	1140 ± 142	t=14.75	<0.001

Baseline comparisons indicated that the intervention and control groups differed significantly in parental stress dimensions, such as sight and sounds, parental role alteration, and total stress scores (Table 2).

**Table 2:** Baseline parental stress by group.

Stress Dimension	Intervention (KC) Mean ± SD	Control Mean ± SD	p-value
NICU sound & noise stress	2.64 ± 1.02	3.42 ± 0.42	< 0.001
Infant appearance & behaviour stress	3.11 ± 1.10	3.30 ± 0.30	0.301
Parental role alteration stress	2.50 ± 1.11	3.44 ± 0.44	< 0.001
Total parental stress score	2.69 ± 1.00	3.38 ± 0.38	< 0.001

A significant reduction in parental stress was observed over time, with parents in the intervention group reporting lower stress, compared to those in the control group, particularly in aspects related to parental role and overall stress (Table 3).

**Table 3:** Changes in parental stress from pre- to post- intervention.

Stress Dimension	Pre (Mean ± SD)	Post (Mean ± SD)	p-value
NICU sound & noise stress	3.05 ± 0.86	19.82 ± 8.24	< 0.001
Infant appearance & behaviour stress	3.21 ± 0.79	59.37 ± 15.54	< 0.001
Parental role alteration stress	3.07 ± 0.90	38.42 ± 10.30	< 0.001
Total parental stress score	3.11 ± 0.77	120.30 ± 28.86	< 0.001

Post -intervention, stress related to sound and noise in the NICU environment did not differ significantly between the two groups, as parents who practised KC reported levels similar to those receiving routine care.

The same pattern appeared for stress associated with the neonate’s appearance and behaviour, where no substantial difference was observed between groups.

In contrast, parents in the intervention group experienced significantly less stress related to disruptions in their parental role, showing a clear benefit from engaging more actively with their neonate. This improvement was also reflected in the overall stress levels, which were lower among parents who participated in the intervention compared with those in the control group (Table 4).

**Table 4:** Post-intervention comparison of parental stress between groups. An independent-samples Student’s t-test was used for the comparison of parental stress scores between Groups A and B. All p-values refer to two-tailed tests, and statistical significance was defined as p < 0.05.

Stress Dimension	Intervention (KC) mean ± SD	Control mean ± SD		p-value
NICU sound & noise stress	19.06 ± 1.72	20.50 ± 2.53	t=-0.72	0.46
Infant appearance & behaviour stress	55.89 ± 5.44	62.50 ± 5.57	t=-1.80	0.11
Parental role alteration stress	32.92 ± 2.89	38.60 ± 6.08	t=-3.36	0.002
Total parental stress score	107.69 ± 9.45	121.40 ± 14.68	t=-2.58	0.015

The intervention group reported a mean overall satisfaction score of 4.14 ± 0.44, whereas the control group had a mean score of 4.18 ± 0.02 (p=0.572). Both groups reported very high satisfaction with NICU care.

Correlation analyses were performed to explore the association between demographic characteristics and parental outcomes. Maternal age demonstrated a consistent negative correlation with all stress dimensions, including total stress (rho=-0.385, p=0.0014), stress related to the neonate’s appearance (rho=-0.389, p<0.001), and parental role alteration (rho=-0.337, p=0.0056), indicating that younger mothers experienced higher stress levels.

A strong positive association was identified between perceived support from relatives and stress related to NICU sounds and noise (rho=0,61, p<0.001), as well as neonate’s appearance (rho=0.73, p<0,001).

Neonate clinical condition, as measured by the ALPS-Neo pain score, showed positive associations with stress related to neonate appearance (rho=0.383, p=0.001) and parental satisfaction (rho=0,473, p<0.001), suggesting that parents of more clinically vulnerable neonates may simultaneously experience heightened stress and value care-related support more strongly (Table 5).

In addition, between-group comparisons revealed that neonates in the intervention group demonstrated lower pain levels and more stable sleep-wake patterns compared to those in the control group.

**Table 5:** Correlations between demographics and parental stress and satisfaction outcomes. Spearman’s rho correlation analysis was performed to assess the relationship between parental characteristics (maternal age, paternal age), neonatal clinical status (gestational age, ALPS-Neo score), and parental stress and satisfaction outcomes (PSS:NICU subscale scores and overall satisfaction). All p-values refer to two-tailed tests, and statistical significance was defined as p < 0.05.

Variable	Stress: Sounds & Noise	Stress: Infant Appearance	Stress: Parental Role	Total Parental Stress
Maternal age (years)	–0.286, p = 0.012	–0.389, p < 0.001	–0.337, p = 0.006	–0.385, p = 0.001
Paternal age (years)	–0.051, p = 0.664	–0.115, p = 0.325	–0.001, p = 0.994	–0.049, p = 0.694
Gestational age (weeks)	–0.102, p = 0.383	0.220, p = 0.056	0.019, p = 0.879	0.110, p = 0.381
Neonate clinical status (ALPS-Neo score)	0.185, p = 0.125	0.383, p = 0.001	0.338, p = 0.006	0.258, p = 0.040

## Discussion

KC has emerged as a central component of family-centred neonatal practise. Originally developed in low-resource settings to maintain warmth and improve survival of neonates, it has since been adopted globally and recognised not only for physiological benefits, such as better thermoregulation, heart and respiratory stability, enhanced breastfeeding, but also for its powerful psychosocial impact on parents and neonates alike [4-7]. By promoting closeness, parental role engagement and early bonding, KC addresses the distorting effects of NICU on parent-neonate relationships and parental emotional well-being. Given the high stress and alienation reported in the NICU, particularly for parents of preterm or medically fragile neonates, KC represents a transformative bridge between high-tech care and human relational healing. The findings of the current study contribute to this body of knowledge by offering novel insights into parental stress, satisfaction and their associations with demographic and clinical variables in a Greek NICU.

Our finding suggests that parents in the KC intervention group exhibited notably lower levels of overall parental stress and stress linked to parental-role alteration compared with those in the routine-care control group (p=0.015 and p=0.002, respectively). While stress related to NICU sounds and noise, and neonate’s appearance did not differ significantly between groups (p=0.46 and p=0.11), interestingly, parents of more clinically compromised neonates reported higher overall satisfaction (p<0.001) and higher stress in certain dimensions (p=0.001). Maternal age showed a protective association - older mothers had lower stress across domains. These results suggest that while KC may not eliminate all NICU stressors, it is strongly linked to parental experience of role engagement and satisfaction, particularly among those facing high clinical acuity.

The protective effect of KC on maternal mental health outcomes is now well- established. A recent Cochrane review found high-certainty evidence that KC reduced moderate to severe postpartum maternal depression and moderate evidence for reduced maternal stress [22]. This aligns with our finding of reduced parental role stress in the intervention group. In terms of parental stress specifically, Zych et al. (2022) found that early and frequent KC was associated with lower PSS:NICU scores in parents of preterm infants [23]. Our results extend this by showing that even in real-life NICU conditions with Greek parents, KC’s main stress modulating effect may be through the parental role domain rather than through all stress domains.

However, the evidence regarding the reduction of parental stress from environmental stressors (sound, alarms, NICU design) remains inconsistent. Our non-significant results for sound/noise stress mirror the broader literature: while KC improves neonate physiological stability and may reduce pain/stress, it does not automatically modify the NICU environment. Pavlyshyn & Sarapuk reported physiological markers of neonate stress decreased after skin-to-skin contact, but did not directly assess parental stress from NICU ambient noise [24]. Thus, the present findings fit within the evidence base and indicate that KC should be viewed as a complementary rather than a standalone intervention for parental stress.

KC also fosters improved parent-neonate interaction and higher parental satisfaction. Yarlagadda, Sandbulte & Downs reported better mother-neonate bonding, increased oxygen and improved parental self-efficacy, all of which underpin enhanced satisfaction scores [25]. The current study’s finding of higher satisfaction among parents of more clinically unstable neonates may reflect this dynamic: when parents feel more empowered through KC in a challenging context, their satisfaction grows. 

The study also found that parents of clinically more vulnerable neonates reported high satisfaction, an observation consistent with findings from qualitative studies, where parents described KC as a tangible way to feel involved in their neonate's recovery, especially when the neonate’s condition is fragile. Similar patterns have been reported by Cai et al. (2024), who found that KC fosters parental optimism and strengthens trust in NICU staff, particularly among families of preterm neonates with respiratory distress [9]. Thus, the trends observed in our study correspond closely to the broader international literature and underscore the notion that KC functions not only as a physiologically beneficial intervention but also as a critical mechanism of emotional support and empowerment for parents during one of the most stressful periods of their lives.

Furthermore, the present findings indicate that neonates in the intervention group experienced significantly lower levels of pain and more stable sleep-wake patterns compared to those receiving routine care. These results are consistent with existing evidence suggesting that KC exerts a beneficial effect on neonatal physiological and behavioural regulation. Skin-to-skin contact has been associated with reduced pain responses during clinical procedures, likely due to multisensory stimulation, thermoregulation, and the modulation of stress-related neuroendocrine pathways [6]. Sleep regulation is a critical component of neonatal neurodevelopment, and the effectiveness of KC on supporting more stable behavioural states seems to have important implications for early developmental outcomes [26].

Despite its strengths, the present study presents several methodological limitations that should be acknowledged when interpreting the findings. The duration of KC intervention was relatively short, approximately 1 hour daily, which does not reflect current international recommendations advocating substantially longer and more continuous KC application. Consequently, the observed effects may underestimate the true potential of KC, as evidence suggests that longer skin-to-skin exposure yields stronger physiological and psychological benefits. The limited duration was influenced by practical and organizational barriers, including parental availability, clinical workload, and NICU operational constraints. The quasi-experimental design and the absence of random assignment may have introduced selection bias, as evidenced by the baseline differences observed between groups.

Additionally, the study was conducted in a single NICU setting, which may limit generalizability due to potentially institutional characteristics, such as staff experience, unit culture, or environmental design. Furthermore, although the study was designed as a pre-post intervention, parental stress was primarily assessed at the end of hospitalization, which may limit the ability to fully capture changes over time and the dynamic nature of parental stress throughout the NICU stay. Finally, the relatively small sample size of 80 parents reduces statistical power, particularly for correlational analyses, possibly obscuring weaker associations, whilst a sample size calculation was not performed, bringing additional limitations of the study.

Overall, the findings related to reduced parental stress due to KC have important implications for NICU practice, highlighting the need for adequate staffing, targeted training programmes, and policies that actively support KC and parental involvement in NICU.

## Conclusions

The findings of the present study provide further evidence supporting the beneficial role of KC in the NICU setting. Parents who participated in the intervention group reported significantly lower overall stress and markedly reduced stress related to parental role alteration, indicating that KC meaningfully strengthens parental involvement and mitigates the emotional strain imposed by the NICU environment. Although parental satisfaction was high across both groups, its positive association with neonatal clinical status underscores the multidimensional factor shaping parental perceptions of care.

The study also highlights the complex interplay between parental demographics, neonatal condition, and psychosocial outcomes. Younger mothers, parents reporting lower support, and those caring for more clinically vulnerable infants exhibited higher stress levels, pointing to subgroups who may benefit most from structured emotional support and enhanced opportunities for KC participation. Despite certain methodological limitations, the results reinforce the growing international body of evidence advocating the systematic integration of KC in neonatal practise. KC appears to offer not only physiological and developmental advantages for preterm and clinically fragile neonates but also substantial emotional and relational benefits for parents. Expanding KC programmes, adhering to updated WHO recommendations, and promoting family-centred care frameworks may significantly enhance the quality of NICU services. In addition, structured staff training, clear clinical protocols, and the establishment of a minimum daily duration for KC are essential to support its consistent and effective implementation.

In conclusion, implementing Kangaroo Care consistently and meaningfully within NICUs has the potential to improve both neonatal outcomes and parental well-being, contributing to a more human, supportive, and developmentally appropriate model of neonatal care. The broader implementation of KC in Greek hospitals appears both feasible and beneficial; however, several practical barriers should be considered, particularly organizational constraints within NICU settings. Addressing these challenges, supportive government and institutional policies may facilitate the successful integration of KC into routine practice in Greece.
